# An evaluation of important plant areas around the world

**DOI:** 10.1111/cobi.70013

**Published:** 2025-03-12

**Authors:** Laura Kor, Fiona Perez, Karen Inwood, Iain Darbyshire, Mauricio Diazgranados

**Affiliations:** ^1^ Department of Geography King's College London London UK; ^2^ Research Department Royal Botanic Gardens Kew Richmond UK; ^3^ Durrell Institute of Conservation and Ecology (DICE) University of Kent Canterbury UK; ^4^ Plantlife International Salisbury UK; ^5^ New York Botanical Gardens Bronx New York USA

**Keywords:** area‐based conservation, IPAs, protected areas, spatial prioritization, tropical important plant areas

## Abstract

Area‐based approaches have long dominated biodiversity conservation and have been reinforced by the Kunming–Montreal Global Biodiversity Framework. The important plant area (IPA) approach is a leading framework for the spatial conservation prioritization of plants and fungi, but over 20 years since its launch, its application and conservation outcomes remained unevaluated. Through systematic mapping and semistructured interviews of key informants, we evaluated IPAs globally. We investigated where and how the framework has been applied, to what extent identification has led to plant conservation, how IPAs are perceived by plant conservationists and researchers globally, and key opportunities and challenges for IPAs. We reviewed over 140 relevant sources, spanning scientific publications, reports, websites, and databases, and interviewed 47 key informants. Most publications focused on developing guidance or identifying IPAs. Sixty‐four percent of informants were aware of IPAs that had been incorporated into conservation processes, with broader benefits of IPAs also highlighted, such as generating botanical data. Overall perception of IPAs was positive; they were seen to provide a unifying focus for plant conservation and as maintaining a flexible and inclusive approach. Opinions were split on the effectiveness of IPA programs in engaging broader stakeholders or incorporating local ecological knowledge. Key themes affecting participant perceptions and lessons learned for bridging the research–implementation gap were found. Informants recommended that IPA programs globally seek more ambitious and targeted funding; tailor stakeholder communications; invest time in cross‐sectoral stakeholder engagement; clarify relationships with key biodiversity areas; and create a single central hub for IPA information. With plants underpinning all terrestrial ecosystems, improved outcomes will have broad benefits for biodiversity protection, particularly as new IPA programs are launched in some of the most biodiverse countries in the world.

## RESUMEN

Los enfoques basados en áreas han dominado durante mucho tiempo la conservación de la biodiversidad y se han visto reforzados por el Marco Global de Biodiversidad de Kunming‐Montreal. El enfoque de áreas importantes para las plantas (IPA, por sus siglas en inglés) es un marco líder para la priorización espacial de la conservación de plantas y hongos, pero más de 20 años después de su lanzamiento, su aplicación y sus resultados de conservación seguían sin evaluarse. Mediante un mapeo sistemático y entrevistas semiestructuradas a informantes clave, evaluamos los IPA a escala mundial. Investigamos dónde y cómo se ha aplicado el marco, hasta qué punto la identificación ha conducido a la conservación de las plantas, cómo perciben los IPA los conservacionistas de plantas y los investigadores a escala mundial, y las principales oportunidades y retos para los IPA. Revisamos más de 140 fuentes relevantes, entre publicaciones científicas, informes, sitios web y bases de datos, y entrevistamos a 47 informantes clave.La mayoría de las publicaciones se centraban en la elaboración de orientaciones o en la identificación de los PIA. El sesenta y cuatro por ciento de los informantes conocían los OPI que se habían incorporado a los procesos de conservación, y también se destacaron los beneficios más generales de los OPI, como la generación de datos botánicos. La percepción general de los OPI era positiva; se consideraba que proporcionaban un enfoque unificador para la conservación de las plantas y que mantenían un planteamiento flexible e integrador. Hubo división de opiniones sobre la eficacia de los programas de los OPI a la hora de implicar a partes interesadas más amplias o de incorporar los conocimientos ecológicos locales. Se encontraron temas clave que afectaban a las percepciones de los participantes y a las lecciones aprendidas para salvar la brecha entre investigación y aplicación. Los informantes recomendaron que los programas de IPA a escala mundial busquen una financiación más ambiciosa y específica; adapten las comunicaciones con las partes interesadas; inviertan tiempo en la participación intersectorial de las partes interesadas; clarifiquen las relaciones con áreas clave de biodiversidad; y creen un único eje central para la información de los IPA. Dado que las plantas son la base de todos los ecosistemas terrestres, la mejora de los resultados redundará en grandes beneficios para la protección de la biodiversidad, sobre todo a medida que se pongan en marcha nuevos programas de IPA en algunos de los países con mayor biodiversidad del mundo.

## INTRODUCTION

Global efforts to protect biodiversity have largely focused on area‐based conservation measures. This was recently reinforced by the Convention on Biological Diversity's (CBD) Kunming–Montreal Global Biodiversity Framework (GBF). Its targets recognize the need for participatory biodiversity‐inclusive spatial planning and aim for at least 30% of the earth's surface to be effectively conserved by 2030 (the “30×30 target”) (CBD, 2022). Numerous methods have been developed to support such targets, with important plant areas (IPAs) representing a leading spatial conservation approach for plants and fungi.

**FIGURE 1:**
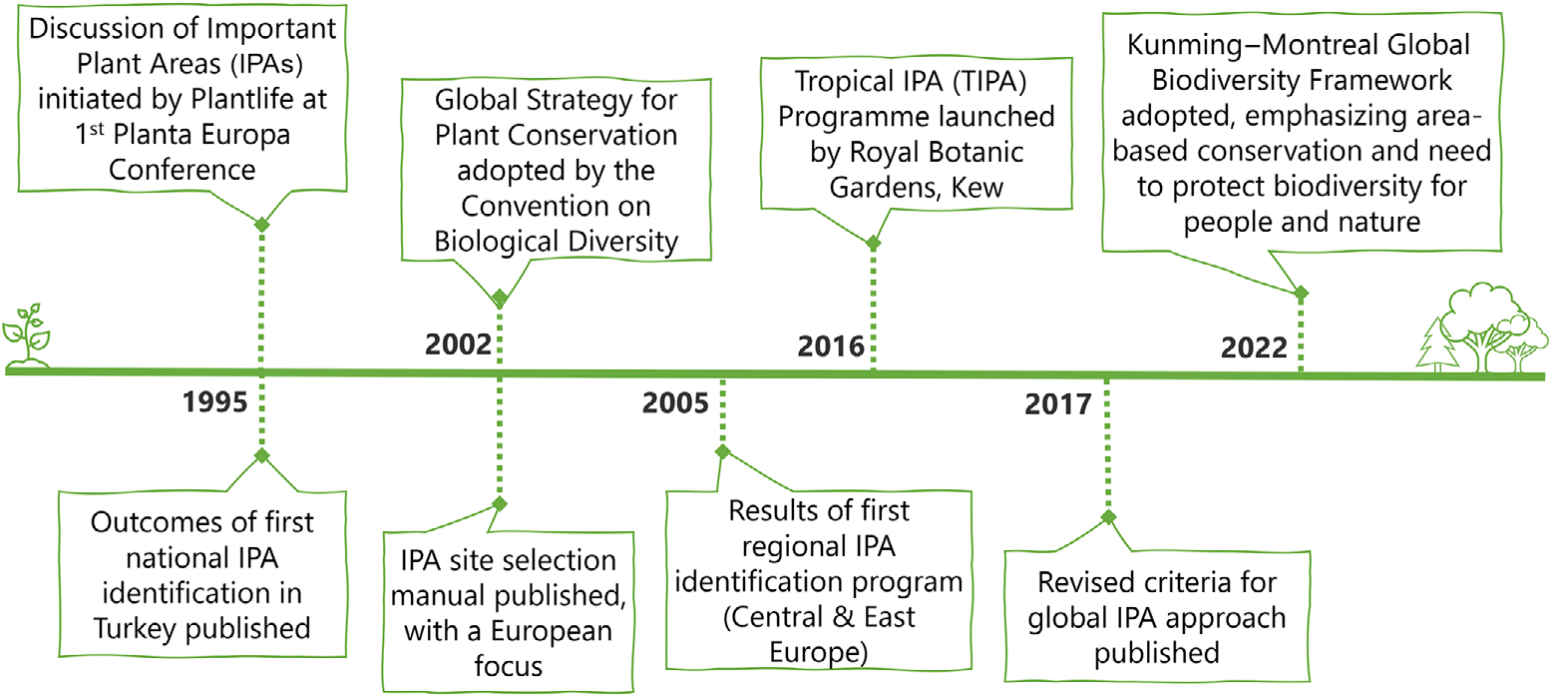
Key milestones in the development of important plant areas.

The IPA concept first emerged in 1995, incentivized by the success of the important bird area (IBA) approach (BirdLife International, 2014; Byfield, 1995; Smart, 1995) (Figure 1). Aiming to establish a network of the best sites for plant conservation throughout the world, IPA guidelines were published in 2002 that contained 3 globally consistent criteria for IPA identification: criterion A on threatened species, criterion B on exceptional botanical richness, and criterion C on threatened habitats (Anderson, 2002). These have been extensively applied, supporting the implementation of the CBD's Global Strategy for Plant Conservation (GSPC) and featuring in the strategies of leading botanical organizations, such as Plantlife International and the Royal Botanic Gardens, Kew (CBD, 2012; Plantlife, 2023c; Royal Botanic Gardens, Kew, 2021). Initial IPA identification and conservation efforts were primarily undertaken in Europe and the wider Mediterranean region, often coordinated by Plantlife. In 2017, the criteria and guidelines were updated (Darbyshire et al., 2017). This coincided with the launch of the tropical IPA program. The new guidelines built on the original European‐focused criteria for a more global approach and incorporated plants of socioeconomic or cultural importance for the first time (Royal Botanic Gardens, Kew, 2016).

The key biodiversity area (KBA) concept also emerged during this time. The KBA Standard unites approaches for the identification of important sites for different subsets of biodiversity (IUCN, 2016). This includes the criteria for IBA and Alliance for Zero Extinction (AZE) being used to inform a standardized methodology and quantitative thresholds. While remaining a separate entity, all AZE sites now also qualify as KBAs (AZE Secretariat, 2024), and many IBAs have been designated as KBA sites.

The updated IPA guidelines recognized the benefits of a single global standard, highlighting the alignment of certain IPA and KBA criteria and the potential for IPA data to feed into plant‐based KBA identification (Darbyshire et al., 2017). However, the IPA approach remains distinct. Reasons include the greater diversity of plants relative to other organism groups requiring different approaches and thresholds. Additionally, in contrast to the KBA Standard's focus on international priorities, the IPA guidelines consider national and subnational conservation assessments and recognize the link between biodiversity and human livelihoods; socially, economically, and culturally useful plants are potential IPA triggers (Darbyshire et al., 2017).

Following decades of coordinated efforts and extensive work on the identification of conservation sites based on IPA criteria, a systematic evaluation of approaches, outcomes, and experiences has yet to be undertaken. Most literature appears to focus on site identification, with little published evidence on how this has or can lead to conservation action and sustainable management. This seemingly reflects the research–implementation or assessment–implementation gap highlighted for other area‐based conservation approaches, such as systematic conservation planning (Adams et al., 2019). However, the goals and methods of each conservation method are distinct.

We synthesized and conducted a critical review of the global IPA approach to answer the following research questions: where and how has the IPA framework been applied; to what extent has identification led to plant conservation action; how are IPAs perceived by plant conservation practitioners and researchers globally; and what are the key opportunities and challenges for IPAs to achieve positive conservation outcomes?

Drawing out key lessons learned from IPAs will help inform future area‐based plant conservation efforts. With plants underpinning all terrestrial ecosystems on Earth, this will have broader benefits for the protection of biodiversity and ecosystem services across the world. We also anticipate that our findings will be of relevance to other global area‐based conservation schemes, such as KBAs and AZEs, and our methods will provide a framework for their evaluation.

## METHODS

To comprehensively review and evaluate IPA application and outcomes globally, we applied 2 approaches for data collection: systematic mapping of IPA literature and semistructured interviews targeting key informants involved with IPAs globally.

### Systematic map

**FIGURE 2:**
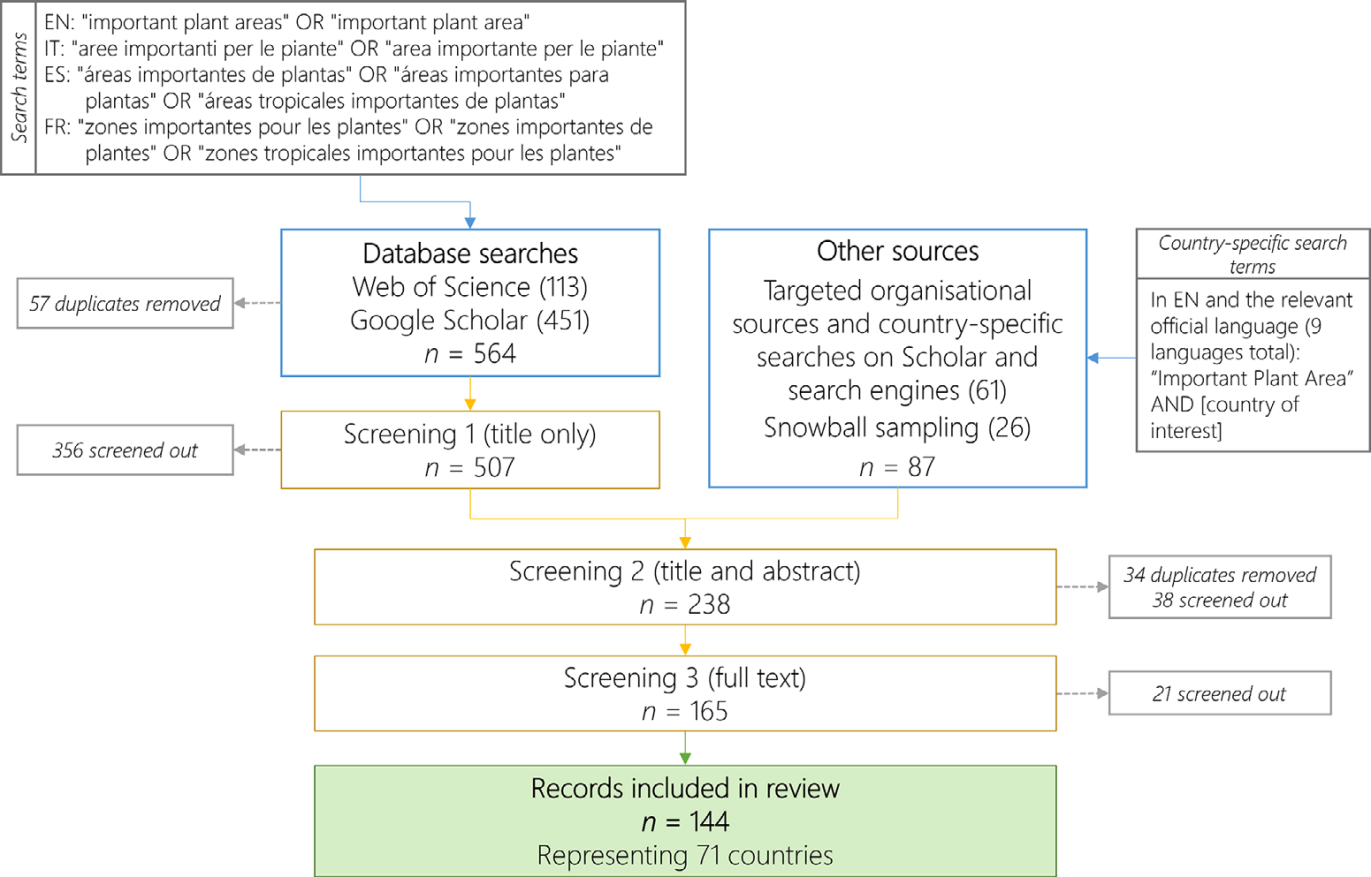
Flowchart of systematic search strategy and screening results for peer‐reviewed and gray literature on global important plant areas (IPA) (EN, English; FR, French; ES, Spanish; IT, Italian).

The method of systematic mapping was developed to collate, describe, and catalog evidence relating to a topic of interest (James et al., 2016)—in this case, IPAs. We searched for relevant publications with the search term *important plant area* in English, Spanish, Italian, and French (Figure 2) in Web of Science and Google Scholar in April 2023. Searches were restricted to items published from 1995 to 2023, and results from Google Scholar were filtered to include a maximum of the 200 most relevant studies for each language. To include relevant gray literature, the organizational websites of Plantlife International and the Royal Botanic Gardens, Kew, were searched. Based on the countries listed on Plantlife's Global IPA Map (Plantlife, 2023b) and Kew's tropical IPA Explorer (Royal Botanic Gardens, Kew, 2023b), targeted country‐specific searches were undertaken in English and the official language of the country in Google Scholar and in the search engines Ecosia and Google (Figure 2). Further sources were identified opportunistically through the citations of studies reviewed and suggestions by interviewees.

Results were imported to Endnote (version 20), and duplicates were deleted. All results were subject to a 3‐stage screening process (Figure 2). Publications in English, Spanish, and Italian were read in the language of publication. Other languages were translated using online document translation services. Although this may have resulted in a less detailed review of texts in certain languages, it was deemed important to include all available sources to reflect the global application of IPAs.

The first stage of screening was based on the title only, the second stage on abstracts (or equivalent), and the third stage on full texts. We applied the following eligibility criteria. For inclusion, sources had to cover methodological approaches or guidelines for IPAs, identification of IPAs, conservation or policy actions related to IPAs, or monitoring and assessment of existing IPAs. Sources were excluded if *important plant area*, or the translated equivalent, was not used in relation to the IPA approach; there was only passing mention of an IPA made as part of taxonomic notes, checklists, and so forth; or the full text was not available. For each publication included in the systematic map, set variables were extracted for analyses (Appendix S1).

### Interviews and surveys

Semistructured interviews were conducted from July to August 2023 and targeted key informants with knowledge and experience of IPAs. Potential participants were identified from authors of relevant publications in the systematic map, members of the Global IPA Network coordinated by Plantlife, recommendations from collaborators, and snowball sampling. We invited 101 people for an interview. We followed the ethics policies of King's College London for human subject research. Participants were presented with an explanation of the purpose and aims in a participant information sheet and provided written consent before the interview. Identities of participants are anonymized in the results (Appendix S2).

An interview guide was created with questions covering the following topics relevant to the study objectives (Appendix S3): participant's background and experience (e.g., sector, involvement with IPAs); approaches to and outcomes of IPA identification; perception of IPA program; conservation action and community engagement; and key opportunities and challenges for IPAs.

Two pilot interviews were conducted to assess the suitability of the interview guide, following which minor amendments were made. Most interviews were by video call; some were delivered in a written format based on participant preference. The interview was designed to be flexible, allowing for the elicitation of specialist knowledge on key topics and for questions irrelevant to the respondent to be skipped. Verbal interviews were recorded and notes were taken. We conducted 47 interviews, primarily in English, with a few in Spanish, that lasted 25–70 min. All responses from verbal and written interviews were collated and curated in Microsoft Excel; answers were anonymized.

### Data analyses

The interview guide included open‐ended and closed questions. Open‐ended, qualitative data were managed and analyzed in NVIVO. We used thematic analysis to assess responses following key principles outlined by Watts (2013) for reading and coding data and selecting extracts for analysis. Pertinent literature and findings from the systematic map were then used to support the discussion and interpretation of interview results.

Closed questions represented participants’ perception of IPA effectiveness through questions presented on a Likert scale. Nominal questions were used to determine conservation action and community engagement. Responses were extracted for data management, analyses, and visualization in R 4.2.0 (R Core Team, 2022). Before combining responses from verbal and written interviews, a Mann–Whitney test was applied, a nonparametric test suitable to compare 2 independent groups with ordinal data (Lepš & Šmilauer, 2020). This showed no statistical difference for any of the Likert scale or nominal questions (*p* > 0.05), which were therefore combined for analyses.

The package likert (Bryer & Speerschneider, 2016) was used to analyze the frequency of rankings and visualize results. We explored the effect of participant background on scores given for each question on IPA effectiveness (with the nonparametric Kruskal–Wallis test [Lepš & Šmilauer, 2020]) and overall perception of IPA effectiveness (with a one‐way analysis of variance fitted to the sum of participant scores). The effect of sector and IPA location were tested as potential explanatory variables for nominal responses to whether IPA identification led to conservation processes or community engagement. We used ggplot (Villanueva & Chen, 2019) to visualize data and tidyverse (Wickham et al., 2019) to manage data sets.

## RESULTS

### Systematic map

**FIGURE 3:**
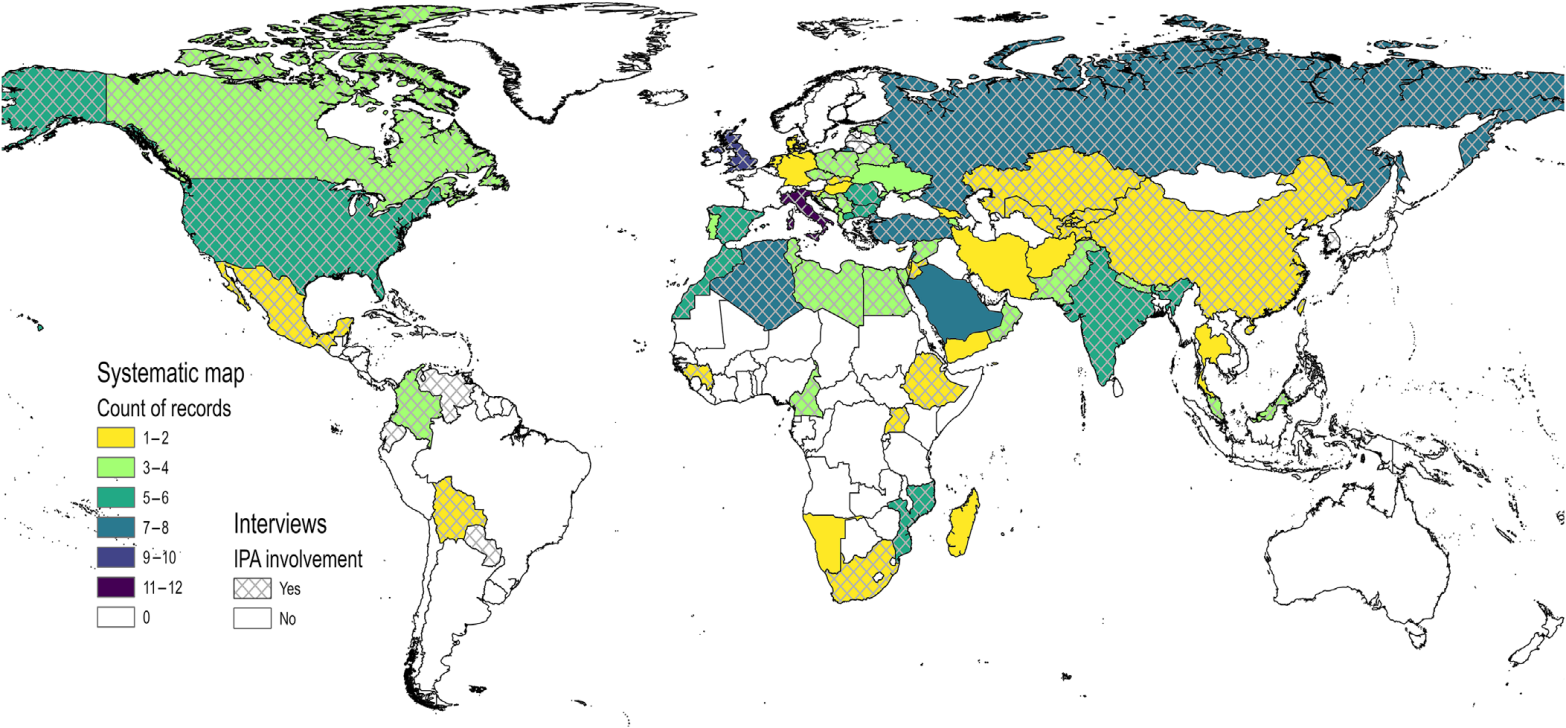
Geographic coverage of publications on important plant areas (IPAs) reviewed and countries where interview participants have been involved with IPAs. Records and interviews are counted multiple times if multiple countries were included, and whole countries are highlighted even if study was relevant at the subnational scale.

A total of 651 results were returned from the sources searched, with 564 from scientific databases and 87 from organizational websites and targeted searches (Figure 1). The screening process resulted in 144 publications being included in the final review. Of these, 119 were in English and the others were in a variety of languages, most commonly French (*n* = 7), Spanish (*n* = 6), and Russian (*n* = 5). Most studies were conducted at the national (*n* = 64) and subnational (*n* = 56) scales (Table 1). Italy was associated with the highest number of publications (*n* = 12) (Figure 3). Overall, and at these scales, most studies focused on IPA identification (*n* = 97). Studies relevant to the global (*n* = 6) and regional scales (*n* = 18) primarily focused on publishing IPA tools and guidance. In line with global IPA guidelines, 105 studies covered plants and fungi in their broad sense, 24 focused on vascular plants only (including studies on specific species or genera), and 12 focused on either algae, bryophytes, fungi, or lichen only.

**TABLE 1:** Categorization of records included in the systematic map of important plant areas (IPAs).

Topic	Category	Count (*n* = 144)
Publication type	Journal article	60
	Report	39
	Web page	14
	Conference proceedings	13
	Book or book chapter	13
	Pamphlet or newsletter	3
	Thesis	2
Study scale	National	64
	Subnational	56
	Regional	18
	Global	6
Language^a^	English	119
	French	7
	Spanish	6
	Russian	5
	Italian	4
	German	2
	Other	3
Continent^b^	Western Europe	46
	Asia	28
	Africa	26
	Eastern Europe and central Asia	26
	North America and Caribbean	11
	Global	6
	Mesoamerica and South America	6
Important plant area focus^c^	Applying criteria for IPA identification	97
	Tools and guidance	44
	Monitoring and assessing	16
	Use in policy and practice	15
	Other	2
Useful plants included	Yes	18
	No	78
	NA	48
Other designations overlapping	Yes	81
	No	2
	NA	60
Conservation interventions	Yes	67 (22 of which mentioned implementation)
	No	49
	NA	28

^a^ Studies published in more than one language counted multiple times.

^b^ Countries assigned to continents based on International Union for Conservation of Nature statutory regions modified to combine south and east Asia and west Asia as Asia. Studies relevant across continents counted multiple times.

^c^ Studies covering different IPA topics counted multiple times.

Regarding conservation action, 67 records (47%) recommended conservation interventions for IPAs. Of these, 22 (15%) reported interventions that had been implemented or attempted (Table 1). In general, implemented actions could be grouped as inclusion in legal or policy mechanisms, awareness raising or community engagement, monitoring activities, or establishment of conservation or sustainable management plans.

Graphical representation of the data showed 2 main peaks in the number of IPA‐related records: the first around 2008–2012 and the second in recent years since 2017 (Figure 1). Regression results showed that, overall, there was a significant increase in the number of records published, with an estimated 7.6% increase per year since 1995 (*p* < 0.05). Western Europe was associated with a significantly higher number of publications than other continents (*p* < 0.05), but that number decreased over time, in contrast with all other continents (full results in Appendices S4 & S5).

A key output of the systematic map was a searchable data set that organized all available records on the identification and protection of IPAs worldwide included in our review. A simplified version of this is available to download from Figshare (https://figshare.com/articles/dataset/Publications_from_Global_IPA_Review/24645360?file=43310670) (Kor, 2023).

### Interviews and surveys

Interviews were undertaken with 47 key informants, 32 via video call and 15 through an equivalent written survey. Most participants were identified through membership in the Global IPA Network or were known to us through our involvement with IPAs (72%). The remaining participants were identified by their authorship of relevant studies in the systematic map or through snowball sampling.

**FIGURE 4:**
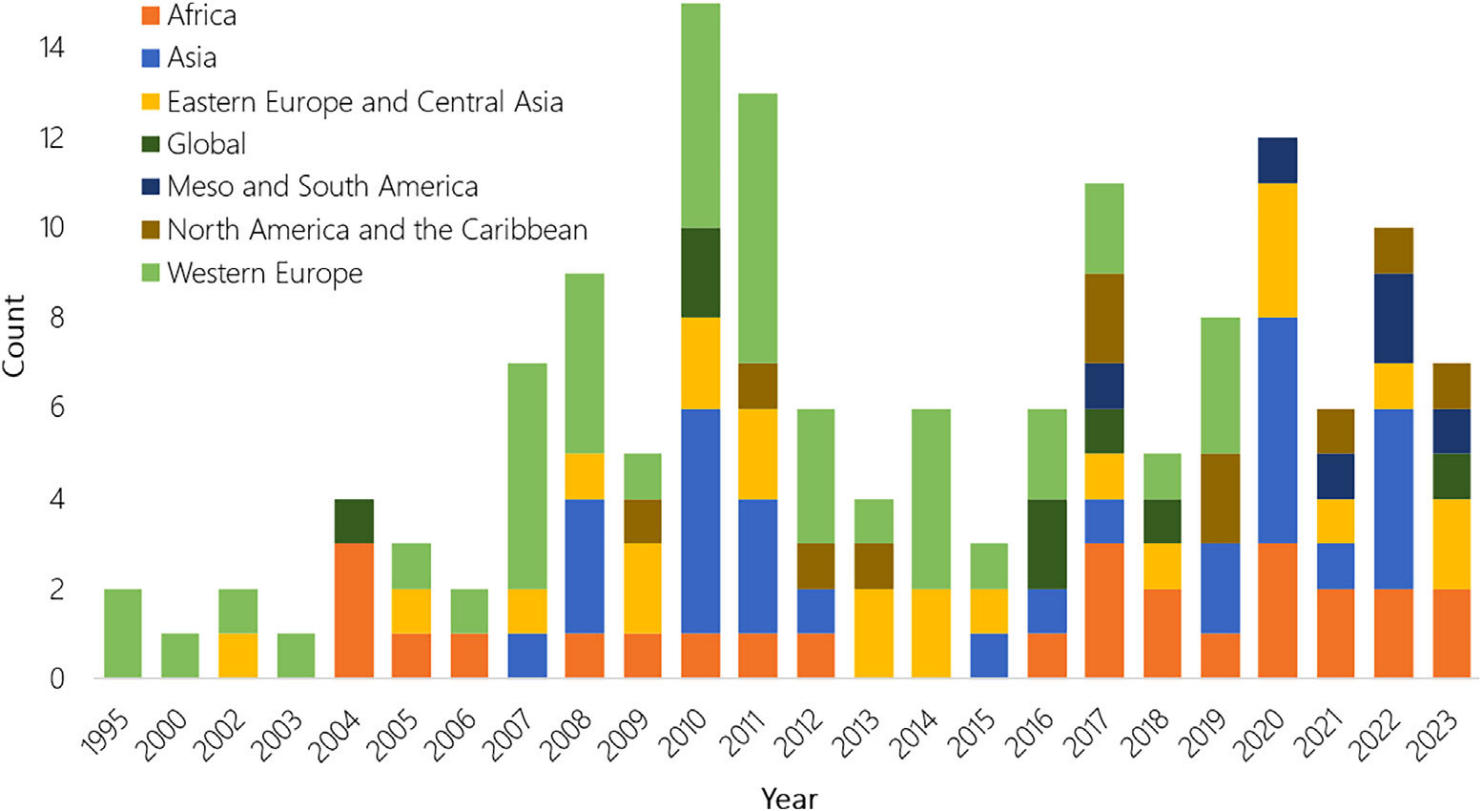
Number of publications per year and location. Countries are assigned to continents based on a modified version of the International Union for Conservation of Nature classifications. Studies in more than 2 continents are counted multiple times.

During the time of their involvement with IPAs, participants worked in research or academia (53%), nongovernmental organizations (NGOs) (34%), government (6%), and other sectors (7%) (including implementation agencies and conservation membership organizations). The majority were based in western Europe (40%) or eastern Europe and central Asia (28%). However, their experience of applying the IPA process was more evenly spread across continents (Figure 4), including eastern Europe and central Asia (36% of participants), Africa (21%), Mesoamerica and South America (13%), western Europe (10.6%), North America and the Caribbean (9%), and the rest of Asia (6%) (remaining 4% of participants were involved with global‐scale policy and conservation).

**FIGURE 5:**
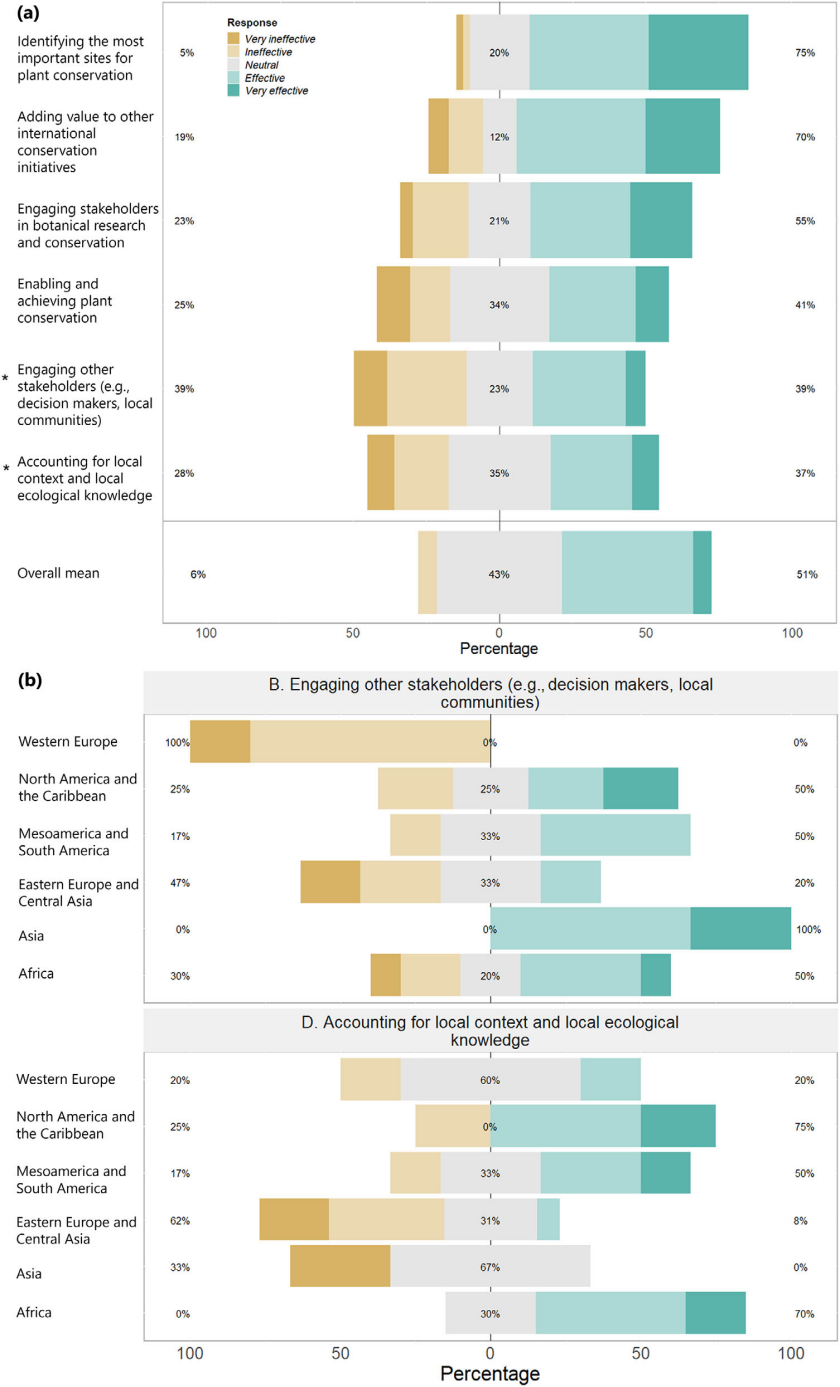
Likert scale ranks for (a) each indicator of important plant area (IPA) effectiveness and (b) indicators ranked significantly differently by participants grouped by continent of IPA involvement (negative scores, IPA very ineffective or ineffective; neutral scores, neither effective nor ineffective [center]; positive scores, IPA effective or very effective).

The mean result across 6 Likert scale questions on IPA effectiveness showed a generally positive perception (Figure 5), but scores varied among indicators. Participants were overwhelmingly positive about the IPA criteria in identifying the most important sites for plants (Question C) (75% of responses were effective or very effective) and adding value to other international area‐based conservation efforts (Question F) (70%). Opinions were more split on their effectiveness in engaging stakeholders beyond research and conservation (Question *B*) (39% of responses effective or very effective vs. 39% of responses ineffective or very ineffective) and accounting for local context and local ecological knowledge (Question D) (37% vs. 28%). Questions B and D were also ranked significantly differently based on the continent in which participants had IPA involvement (*H* = 11.8, *p* < 0.05 for B; *H* = 16.2, *p* < 0.01 for D). Question B on effectiveness in engaging broader stakeholders was viewed most negatively by those with experience applying IPAs in western Europe (100% of responses negative), and question D, on incorporating local knowledge, had lower scores from those who had worked in eastern Europe and central Asia and the rest of Asia (Figure 5).

Most participants reported that they knew of examples where IPAs were incorporated in conservation designations or other in‐country conservation processes (*n* = 30; 64%). Twenty‐three participants (59%) were aware of local IPA conservation action or community engagement. Although the former were not related to location or sector, participants from the NGO sector were significantly more likely than other groups to be aware of community engagement (*H* = 9.7, *p* < 0.05).

Qualitative data revealed a wide range of reasons for the scores assigned and answers given. Across the effectiveness indicators, 8 key themes emerged from inductive coding. When discussing the incorporation of local ecological knowledge, stakeholder engagement, enabling conservation action, and conservation outcomes, the most common themes were linked to existing conservation agendas (relevant at the national and global levels with IPA impact dependent on the number of competing conservation initiatives); NGO and civil society involvement (often highlighted as crucial for continued momentum and successful conservation and engagement outcomes); tailored communication to the stakeholders in question; and capacity of initial identification team (importance of community engagement widely acknowledged, but skills and institutional aims of participants from the research and academic sector often not able to meet this).

In identifying the best sites, adding value to other global initiatives, and engaging stakeholders in botanical research and conservation, the most common themes included the IPA program's central goal (seen to provide a simple message and unifying global focus for plant conservation); data availability and data sharing (highlighted in terms of data gaps for identification and a need for a centralized database on existing IPAs); flexibility complexity, and standardization (views contrasted on the ease of IPA criteria application and whether they should be more closely unified with other global initiatives); and breadth and inclusivity (provided through the IPA criteria incorporating broad taxonomy; ecological scales; and social, economic, and cultural values).

Interview participants were asked to describe what they saw as key opportunities and challenges for IPAs to support plant conservation. The factors that emerged from coding responses to this query were grouped into 5 key themes: strategic context, stakeholder engagement, guidelines and criteria, postidentification momentum, and knowledge and capacity.

## DISCUSSION

IPAs are a leading global area‐based conservation approach for plants and fungi and support targets of the CBD's GSPC. Before our research, Plantlife International's Global IPA map listed 52 countries with IPA identification completed or in progress (Plantlife, 2023a). However, as with other similar global initiatives, details were dispersed and a systematic effort to establish key lessons learned was lacking.

Through systematic mapping and semistructured interviews, we found that 76 countries had engaged with the program to date, either at a national or subnational scale and sometimes as part of regional programs. We used our research questions as the focus of the “Discussion.” The findings should be seen within the limitations of this study's scope, where all participants already had some level of involvement with the IPA program, and the evaluation of effectiveness was based on publications and self‐assessment rather than independent analysis of IPA sites.

### Application of IPAs

The systematic map showed the wide geographical application of IPAs. This was reflected in interview participants’ view that the program's clear central aim provided a unifying focus for botanists and helped to raise awareness of plant conservation. One participant explained: “the global vision gives the real value … I've been working on plants and botany for the last 25 years and I think it's the only project that really puts plants in front of everything.” However, not all participants shared positive views on IPAs’ ability to raise interest. Some were concerned that few people beyond those directly involved are aware of the scheme, and some highlighted persistent plant blindness within and beyond the conservation community.

Most of the 97 records that centered on IPA identification followed the European‐focused guidelines published by Anderson (2002) or the revised global guidelines by Darbyshire et al. (2017). The ability of these criteria to effectively identify the best sites for plant conservation was scored positively (Figure 5). This was largely attributed to their basis in sound scientific principles, flexibility, and inclusivity (taxonomic breadth, incorporation of national priorities, approaches at species and habitat levels, and inclusion of plant uses). However, some participants mentioned struggling with “cumbersome” criteria application. This was more common among those working independently of projects coordinated by Plantlife or Kew. A participant working on IPA identification in South America called for stepwise instructions and code for data analyses to be published and for a greater exchange of knowledge between countries to be facilitated. There was also some confusion surrounding the distinction between IPAs and tropical IPAs. However, a participant involved in establishing the tropical IPA program explained that tropical IPAs are increasingly viewed as falling within the wider Global IPA Network and that projects are dropping the *T*, as in recent publications (Darbyshire et al., 2023; Murphy et al., 2023).

Two gaps recurringly highlighted in IPA guidelines were how to define site boundaries and how to prioritize sites. The latter was a particular problem in high‐biodiversity areas. A participant in South Africa explained that “in our country, we have thousands [of IPAs]…we've had to decide that [prioritization] ourselves.” This was reflected in literature, where records classified as tools and guidance commonly designed scoring criteria or amended IPA thresholds to fit their national or regional context (e.g., Alves et al., 2016; Bou Dagher‐Kharrat, 2018; Hamidah et al., 2020). For the delimitation of site boundaries, the IPA guidelines contain only high‐level suggestions, contrasting with the KBA Standard, which dedicates a section to this (IUCN, 2016). However, because global generalizations can be hard to apply in site‐specific contexts, numerous participants conversely highlighted the flexibility of the IPA approach as a key benefit.

Literature on IPA identification and interview participants also commonly mentioned data limitations and that occurrence records are often biased toward specific locations or taxonomic groups (e.g., Kor & Diazgranados, 2023; Yahi et al., 2012). In many cases, this was overcome through strong reliance on relevant experts in the identification process (e.g., Chamchumroon, 2008; Radford et al., 2011). South Africa provided an interesting case study in which a lack of data previously prevented IPA identification (von Staden & Lotter, 2015), but the huge growth in biological records through citizen science initiatives made this identification possible.

The literature we reviewed commonly listed climate change as a threat to IPAs and their features (Hamilton, 2008; Montana Plant Society, 2023; Plantlife, 2021). This is a problem for all area‐based conservation approaches; the static boundaries of protected areas are at odds with the dynamic response of species to climatic changes (Hannah, 2008). However, this was raised by only one interviewee, who suggested the need to move toward concepts of functional habitats and work more closely with horticulturists and ex situ conservationists. Greater consideration of functional diversity in conservation prioritization has been more broadly suggested in conservation literature; however, estimating functional and phylogenetic metrics is more complex than species‐based measures and would inevitably involve trade‐offs (Cadotte & Tucker, 2018).

### Changes through time

There was an overall increase in the number of IPA‐related sources published per year since 1995. However, this increase has not been consistent (Figure 4; Appendix S5). The first peak (2010–2011) represented an initial wave of IPA identification projects and outputs across Europe and the Mediterranean region, often coordinated by Plantlife. The latter peak (2017 to date) followed the launch of the tropical IPA program by Kew in 2016 and the publication of new global guidelines in 2017 (Darbyshire et al., 2017). This also explained the geographic shift of publications through time, from an initial focus on Europe to other continents.

More subtle changes were revealed in interview responses. One of the main updates in the 2017 guidelines was the inclusion of socially, economically, and culturally important plant species as potential triggers under IPA criterion B (botanical richness) (Darbyshire et al., 2017). This seems to have vastly altered participants’ perception of the effectiveness of IPAs in incorporating local ecological knowledge. Participants from European countries (who applied the original 2002 criteria) overwhelmingly scored this indicator negatively, in contrast with positive scores from respondents in the rest of the world (largely undertaken since 2017) (Figure 5). This shift reflects wider advances toward socioecological approaches in conservation (Virapongse et al., 2016) and was linked to the expansion of IPA application to the Global South. A participant involved in developing the updated guidelines explained, “it's really important for us working in the developing world, as species which are important for conservation is such a rarefied concept … But once you start talking about conserving socioeconomically important species … people are really gripped by that.”

Despite this enthusiasm, challenges to incorporating local context and knowledge were highlighted, with a major factor being a lack of capacity and relevant skills. First, those leading the identification process are primarily botanical experts with limited community engagement experience. This makes it difficult to gather plant use data in a sufficiently systematic manner to inform IPA identification, demonstrating that even with the best intentions the nature of area‐based conservation hinders the incorporation of different knowledge systems (Wyborn & Evans, 2021). Second, there is often continued reliance on international expertise. A participant from Mozambique explained: “I understand that the criteria were developed to account for local context, but in some countries, this is challenging … In my opinion, if the process is not 100% led from the national level, then there will be some distortion of the local context. This is not just about the criteria per se but the reality of some countries.” In light of this, ongoing efforts at capacity building were mentioned, with many tropical IPA projects including training and in‐country capacity building (e.g., Royal Botanic Gardens, Kew, 2023a). Following the earlier IPA‐Med project (Radford et al., 2011), a botanist in Tunisia mentioned subsequent training of university students: “we learnt [conservation skills] from the training we got on … projects starting with IPA identification, so we try to transmit this knowledge.” Participants also highlighted the ability of IPAs to interest early career scientists in plant conservation.

### From identification to conservation action

Academic literature on other area‐based approaches, such as systematic conservation planning, is often skewed toward methodological studies and provides little evidence on conservation outcomes (McIntosh et al., 2018). Results from our systematic map indicated the same trend in IPA literature. Most sources focused on identification, and only 15% reported conservation interventions (Table 1). This contrasted with interview results, in which 64% of respondents knew of examples of IPAs being incorporated into conservation designations or other in‐country conservation processes. There are various potential explanations for this difference. First, publications tend to be led by authors from research institutions, who are more likely to be involved in the data‐heavy step of IPA identification, whereas resulting conservation action is more likely to be led from NGO and policy spheres, where publication is not an institutional priority (Sinclair et al., 2018). Second, potential participants contacted may have been more likely to consent if they had a more active engagement with the IPA process, potentially skewing interviewees toward those involved in IPA conservation outcomes.

Similar types of conservation interventions were highlighted by literature sources and interviewees. However, the latter mentioned them in more countries and in greater detail. We grouped the types of conservation action found to arise from IPAs into 4f categories: legal protection, nonstatutory policy and management, community‐based conservation, and generation of data and knowledge.

### Legal protection

Statutory protection of IPAs was most common in eastern Europe, where they informed the identification of Natura 2000 sites, a network of protected areas designated under the European Union's Habitats and Birds Directives (EEA, 2023). Therefore, despite no longer always being referred to as IPAs, many sites are under legal protection in, for example, Croatia and Macedonia (Radford & Ode, 2009). One participant mentioned that the central and eastern Europe IPA project “helped transition countries show they had a conservation network.” However, in western Europe, other protected area systems were established before IPA identification. A participant in Italy explained that in attempting to engage political stakeholders in IPAs “most of the time it was ‘we don't need it, what is it for? We already have Natura 2000 or other protected areas’,” a sentiment closely echoed by Spanish respondents.

This contrast highlights the importance of existing conservation agendas. The IPAs were often seen as superfluous by stakeholders in countries with numerous existing conservation approaches but were thought to support biodiversity goals in some areas with less history of spatial conservation planning. This was also raised at the global scale in relation to the broader recognition of KBAs compared with IPAs. Beyond Europe, Guinea and Bolivia provided examples of tropical IPAs that have been or are being incorporated into protected area systems. A key enabling factor highlighted was preexisting or early and persistent involvement of stakeholders, including political stakeholders and in‐country NGOs.

### Nonstatutory policy and management

Although some participants believed a lack of automatic legal protection meant IPAs were ineffective for conservation action, others saw this as a strength: “in the UK, I see it almost more of an opportunity… as we already have a lot of … protected land. The issue is getting them better managed.” An example of IPAs influencing policy and management without statutory protection was provided by the British Virgin Islands and the Province of Saskatchewan, Canada, where IPAs are factored into planning decisions through inclusion as a layer in the GIS portals of relevant authorities. Additional uses of IPA spatial data include combining it with other environmental criteria to develop sustainable management advice for large commercial landowners (conservation NGO in Turkey) and using it to inform ex situ conservation strategies through targeting seed collection in multiple countries.

Management interventions were more easily enabled when IPAs overlapped with existing conservation sites, with site management plans providing a clear means through which plant species conservation could be established. There were also examples of IPA inclusion in regional conservation strategies. One interviewee highlighted the Montana Plant Conservation Strategy (USA), and a reviewed paper mentioned the inclusion of important fungi areas in the Tuscan Biodiversity Action Plan (Italy) (Perini et al., 2014).

### Community‐based conservation and NGO involvement

The community‐based conservation initiatives mentioned took a range of forms, from passive engagement via talks and leaflets to active volunteer networks for monitoring IPAs and their species (e.g., Murphy & Richards, 2022; Sanchez et al., 2019). In some cases, community conservation highlighted in literature was revealed to be inactive by interviewees, such as the IPAnet project in Turkey (Obanet, 2015). Most examples of conservation in the systematic map were in sources other than peer‐reviewed articles (i.e., reports, web pages, and conference proceedings), contrasting with the overall characteristics of the literature reviewed but reflecting the fact that such initiatives were significantly more likely to be mentioned by interviewees from the NGO than research sector.

The importance of in‐country NGO involvement was most clearly voiced by a participant from Macedonia, the country with perhaps the longest running example of active community‐based IPA conservation. Although IPA identification (Radford & Ode, 2009) and the subsequent Natural Networks of Places and People project (Plantlife, 2013) were undertaken over a decade ago, the interviewee described ongoing plant monitoring and lobbying for IPA site protection by civil society groups, adding that “IPAs…is something they're proud of…[local partners] are now doing the job by themselves.” Crucial to building this momentum was the work of individuals from the Macedonian Ecological Society: “connecting with lots of people…visiting people who lived or worked in IPAs… You stay in contact with one person who is passionate about [conservation], he finds another one….a really spontaneous snowball effect and a continuation of that collaboration.” The importance of persistence was also stressed by a participant in Tunisia, who shared that efforts to change local agricultural practices to benefit endemic plant species were successful only after “many, many sessions with raising awareness.” In the United Kingdom, IPA community engagement has largely continued to be led by the NGO Plantlife, often through more formal projects. For example, community engagement activities and wider awareness campaigns following IPA identification led to much greater recognition of the temperate rainforests of the British west coast, in turn enabling alliances with other conservation NGOs and further increasing overall impact and awareness.

Meanwhile, for tropical IPAs, one participant explained: “as a research institute we are judged more on our [academic] outputs, and working more with stakeholders would take time… The most important thing to allow for this is engaging with socioeconomic NGOs.” In Bolivia, such collaborations have led to the establishment of value chain networks and the ongoing development of a certification scheme to support conservation‐through‐use approaches involving Indigenous and local communities. A similar outcome was achieved in the Himalayan region of China, part of the first‐ever IPA identification effort based on useful (specifically medicinal) plants (Hamilton & Radford, 2007). By connecting local communities and international traders, an income source from cultivated medicinal gardens was generated, which supported wild plant conservation. An interviewee described how the project spread to other nearby communities and is “…still continuing. Conservation is not a one‐off thing; you need to do it forever.” In Guinea, environmental education is a core element of the tropical IPA project's community‐based efforts, including the publication of a booklet for schools (Cheek, 2017) and the recruitment of a dedicated in‐country environmental officer for outreach. In response to requests from local collaborators, community tree nurseries were established to grow useful plant species, thereby minimizing the wild collection of threatened plants from IPAs. These interventions require long‐term strategies. One participant explained that “we're hoping that in the following years, we can grow enough plants that the communities can sell to reforesting projects…to give a revenue.”

### Generating data and knowledge

In addition to directly influencing conservation plans and projects, IPA efforts have generated, mobilized, and increased access to plant data. Many IPA literature sources described extensive botanical data collection through targeted fieldwork and the collation of previously disparate information in literature, herbaria, and databases. It was beyond the scope of our study to assess how often these data have been made publicly available. However, they have substantially increased knowledge of the ecology and distribution of plants and fungi across many countries, thereby supporting target 21 of the GBF to ensure that data and knowledge are accessible to a range of stakeholders. This has enabled the production of verifiable checklists, species red lists, habitat maps, and new scientific descriptions of species (e.g., Couch et al., 2019; Khennouf et al., 2018; Radford & Ode, 2009), but the need to create a centralized repository for such data was highlighted by several interviewees. Other conservation‐related work has also been mobilized by IPAs. Participants from several countries described IPA identification as the natural next step following the national red listing of plants (del Valle et al., 2003); the IPA criteria provide a standardized means to move from species extinction assessments to area‐based conservation.

### Opportunities, challenges, and recommendations

Although interviews succeeded in highlighting more IPA conservation examples than literature, the examples often focused on one or a few sites, indicating that many identified IPAs have not directly led to documented conservation outcomes. This was often attributed to a lack of resources, support, and momentum postidentification. Even in Italy, the country with the most IPA‐related literature and ministerial support for IPA identification (Blasi et al., 2009), support has dwindled. A respondent said, “after 5 years there was no more money and people have to find money.” Although community‐led efforts for IPAs in Macedonia continue, they involve only a handful of sites, chosen as pilots directly after identification. Therefore, although IPA identification continues to increase globally, there is a way to go to truly achieve the other crucial half of its primary aim to “identify and protect a network of the best sites for plant conservation” throughout the world (Anderson, 2002) and to meet GSPC targets.

This gap between identification and conservation action is common among area‐based initiatives. The assessment–implementation gap has been highlighted for systematic conservation planning (Adams et al., 2019). There is some evidence of the usefulness of KBAs in identifying sites of biodiversity significance (Plumptre et al., 2023), but a comprehensive evaluation of subsequent conservation outcomes is lacking. Literature on ecologically or biologically significant marine areas (EBSAs) indicates that important areas for marine biodiversity have been identified, but few examples exist of resulting conservation action (Harris et al., 2022).

There are therefore clear challenges associated with moving from spatial conservation prioritization to conservation action (Adams et al., 2019; Harris et al., 2022; McIntosh et al., 2018). However, with the scale of global biodiversity loss, shifting priorities in conservation approaches, and momentum gained since the launch of the tropical IPA program, there was a sense among participants that this is a time for IPAs to gain policy recognition and contribute to plant conservation. Drawing on themes from this study, alongside key challenges and opportunities highlighted by participants, we devised 5 key recommendations for IPAs: provide more ambitious and targeted funding, tailor stakeholder communications, time stakeholder engagement properly, clarify the relationship of IPAs with KBAs, and provide a single central hub for IPA information. Some of these are relevant at the global scale for the overall IPA program approach, and others are more appropriate at the project scale.

Funding should be more ambitious and targeted. Many participants highlighted resources as a key challenge for IPAs, and the need for more ambitious funding goals was raised across scales. At the project scale, funding is required to make identification as comprehensive as possible, enable engagement and conservation action postidentification, and update identified networks with new data. To address this, tropical IPA participants mentioned the ability to attract philanthropic funding when conservation, livelihood, and capacity‐building outcomes were clear. Such an approach also encourages conservation action to be integrated into the initial aims and planning of IPA identification, increasing their likelihood of success (Adams et al., 2019; Sinclair et al., 2018). At the global scale, increased resources are required for a more strategic approach and to support lobbying for greater attention in conservation policy. The need for greater ambition in leveraging large grants was raised, such as from philanthropic commitments and closer engagement with the Global Environment Facility. The people‐centric focus of IPAs was also highlighted as a differentiator from other conservation efforts in gaining support from these sources.

Stakeholder communications should be tailored to match the specific goal and audience. Communication challenges related to engaging political, community, and industrial stakeholders were repeatedly raised. This was relevant to conveying the importance of plant conservation, sharing the outcomes of IPA identification (“people on the ground aren't interested in maps”), and differentiating IPAs from traditional protected area management (“as soon as you start talking about areas for conservation, there is a level of defensiveness”). To engage stakeholders, there is a need to highlight benefits of specific relevance to them. The potential power of focusing on useful plants and more broadly placing IPAs in an ecosystem services frame was highlighted. However, this should be location specific. Adams et al. (2019) highlight the importance of considering the relevant socioecological context in the planning and identification stage of conservation areas. To convey the outcomes of IPA identification, messages should be streamlined based on the intended audience. For community engagement, relevant and engaging messaging can generate pride in local environments. For national and local decision makers, a participant suggested that there should first be coordination among the conservation community to bring together different spatial conservation planning initiatives for simplified policy recommendations. Drawing on the Colombian context, they explained: “there are more than 50 exercises on spatial planning…How do we translate this to stakeholders? … This needs to be solved by the institutions leading the initiatives.”

Stakeholder engagement should happen early and be sustained. This was highlighted as key to IPA projects that successfully generated political support. Sustained engagement included involving relevant authorities in meetings during initial identification steps and continued conversations on how IPA outputs could feed into decision‐making. Such approaches would facilitate the assimilation of an implementation strategy in the IPA identification phase (Adams et al., 2019). To realize the potential of IPAs in protecting threatened plants for humans and engaging local communities, there is a need to demonstrate long‐term commitment to local areas. This reflects the fact that relationships of trust are fundamental in achieving community‐enabled conservation because they encourage the coproduction of knowledge and solutions rather than viewing communities as a focal point for input (Armitage et al., 2020).

The relationship between IPAs and KBAs should be clarified. Although most participants thought that IPAs add conservation value beyond other global initiatives, many acknowledged the broader recognition that KBAs have in policy spheres. A global spatial comparison between KBAs and IPAs is lacking, but existing analyses indicate that sites often do not overlap; 60% of IPAs in the Mediterranean do not overlap with KBAs (Radford et al., 2011). One participant described closer standardization of the programs as “the biggest, easiest opportunity for IPAs.” This does not necessarily mean losing IPAs as a distinct framework; rather, it means aligning IPA and KBA efforts where the criteria converge and still maintaining the distinguishing elements of IPAs. As a participant involved in IPA conception voiced, this would prevent duplication of efforts and provide “a single message to decision makers…. It's not what's best for IPA, KBA, RAMSAR, etc., but how we can harmonize them to protect biodiversity.”

The need for simplification and standardization of the IPA approach itself also emerged. This was related to clarifying differences between IPAs and tropical IPAs, for which steps have already been taken. More importantly, the need for one centralized platform to house all IPA‐relevant guidelines, information, outputs, and data was reiterated by multiple participants. Although we attempted to begin this process, a key next step would be to make all data on identified IPAs accessible and downloadable online. The Tropical IPA Explorer portal (Royal Botanic Gardens, Kew, 2023b) and IPA Database (Plantlife, 2023b) are good starting points, but unifying these and making them more comprehensive could increase transparency, encourage more academic engagement, and support authorities in conservation planning. Furthermore, IPA data could be integrated into broader global databases for conservation areas, such as Protected Planet (UNEP‐WCMC & IUCN, 2023). This would support more cohesion between area‐based conservation approaches and wider recognition of IPAs in conservation and planning decisions.

We found that IPAs have contributed to plant conservation efforts in many ways, from informing protected area designations to the planting of community tree nurseries. This suggests that IPA practitioners are actively seeking to achieve conservation outcomes through the identification of priority areas for plants. However, our results also add to existing evidence of a research–implementation gap in spatial conservation assessments. The lessons learned and recommendations made are timely as IPA application continues to grow with new national programs launched in some of the most biodiverse countries in the world. These lessons are also of relevance to other area‐based conservation approaches, as the conservation community seeks to meet the targets of the GBF while delivering effective and equitable outcomes for people and nature.

## Supporting information



Supporting Information
