# Supplementary material for: An evaluation of important plant areas around the world
Source: Conserv Biol. 2025 Mar 12;39(4):e70013. doi: 10.1111/cobi.70013 (PMC12309633; doi:10.1111/cobi.70013)

**Supporting Information**

**An evaluation of important plant areas around the world**

L. Kor, F. Perez, K. Inwood, I. Darbyshire, M. Diazgranados

**Appendix S1:** details on the variables and categories mapped in the systematic map

| **Theme** | **Variable** | **Categories (where applicable)** |
| --- | --- | --- |
| Bibliographic details | Authors |  |
|  | Title |  |
|  | Publication year |  |
|  | Publication type |  |
|  | Journal |  |
|  | Language |  |
|  | Lead organisation type | Academic / Research  Public body  NGO  Government  Public body / Implementation agency  Other |
| Study location | Study country |  |
|  | Study region |  |
|  | Study continent | *Defined based on IUCN statutory regions, but combining South and East Asia and West Asia as "Asia"*  [*https://portals.iucn.org/library/sites/library/files/documents/2022-002-En.pdf*](https://portals.iucn.org/library/sites/library/files/documents/2022-002-En.pdf) |
|  | Study scale | Global  Regional  National  Sub-national |
| Key aims and findings | IPA focus | Tools and guidance  Applying IPA identification  Monitoring and assessing  Use in policy and practice  Other (xxx) |
|  | Publication aim |  |
|  | Taxonomic focus | Multiple  Vascular plants  Vascular plants (xxx)  Fungi  Bryophytes  xxx |
|  | IPA identification | Y  N |
|  | Useful plants | Y  N |
|  | Details on useful plants |  |
|  | IPA guidelines | Y  N |
|  | Guideline summary / differences from global |  |
|  | Key findings |  |
| Conservation-related | Other designations | Y  N |
|  | Threats |  |
|  | Conservation intervention suggested and/or attempted | Y  N |

**Appendix S2**: Participant Information Sheet

**Informed consent for participation in *Review of Important Plant Areas (IPAs)***
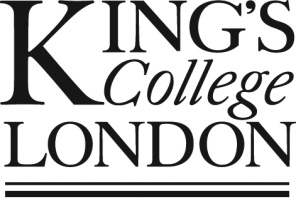


Ethical Clearance Reference Number: LRS/DP-21/22-22709

Date: 16 June 2023

I am a PhD student at King’s College London and the Royal Botanic Gardens, Kew and would like to invite you to participate in an interview which forms part of my research. Before you decide whether to take part, it is important to understand why the research is being done and what it involves. Please read the following information and let me know if you have any questions.

**What is the purpose of this project?**

My research focuses on the conservation of useful plants, including the application of IPAs. While the main geographical scope of my PhD is in Colombia, my final chapter consists of a review of global IPAs, in collaboration with Plantlife. Through a literature review, interviews, and focus groups, this aims to answer the following questions:

1. What methods have been applied to, and results gained from, IPA identification to date?
2. To what extent have IPAs involved useful plants and how has this been approached?
3. To what extent has IPA identification led to plant conservation action and outcomes?
4. How is the IPA approach perceived by plant conservationists and researchers?

**Should you participate in this research?**

You have been invited to participate due to your experience of the IPA approach. Your participation is completely voluntary, and it is your personal decision whether to take part, with no financial compensation offered. You can withdraw from the research at any time while it is ongoing. We invite you to partake in the following activit(ies):

- Interview via video call (or written interview if preferred) related to experience and perception of the IPA approach (30-60 mins). Topics to be covered, with example questions:
  1. Experience and involvement with IPAs
  2. Approaches to and outcomes of IPA identification

*e.g., “what IPA criteria did you apply?”*

- 1. Perception of IPA programme

*e.g.,* “*Based on your experience, on a scale of 1 to 5: How effective are IPAs in engaging stakeholders working in botanical research and conservation?*”

- 1. Conservation action and community engagement

*e.g., “Have the IPAs you have been involved with been incorporated into conservation designations or processes in country?”*

- Potential follow-up online focus group with other members of the IPA network (approx. 2h, with 6-8 participants). Topics to be discussed:
  1. Strengths and weaknesses of the IPA approach
  2. Lessons learnt and case studies
  3. The future of IPAs in global plant conservation

If agreed to by participants, interviews and focus groups will be recorded to help facilitate accurate notetaking and data processing. Please confirm in the consent form and/or at the start of the interview or focus group if you consent to being recorded.

**Data Handling and confidentiality**

Your data will be processed under the terms of UK data protection law (including the UK General Data Protection Regulation (UK GDPR) and the Data Protection Act 2018). If you would like more information about how your data will be processed under the terms of UK data protection laws please visit: <https://www.kcl.ac.uk/research/support/research-ethics/kings-college-london-statement-on-use-of-personal-data-in-research>

Information collected will be used for scientific publications, presentations, thesis reports, and online articles. If consent is given, audio and/or video from the interviews will be recorded on Microsoft Teams and transcribed using their in-built transcription service. Please keep information shared by other participants in focus groups confidential and be aware that confidentiality of information you share in such groups cannot be guaranteed.

The data recorded through consent forms, interviews and focus groups will be pseudononymised after collection. This means that names will be removed but it may be possible to link information back to a participant e.g., through the country of involvement with IPAs. Please indicate in your consent form if you request your data to be completely anonymised (information on your geographic location, organisation and any other potentially identifiable data will be removed from interview responses).

Data collected will only be shared within the research team, including collaborators at Plantlife. Data sharing will be done through a secure folder hosted on Microsoft SharePoint and data will be stored on SharePoint until the completion of my PhD (December 2023). Data may be stored for longer if new related research projects are developed.

King’s College London has a responsibility to keep information collected about you safe and secure, and to ensure the integrity of research data. Specialist teams within King’s College London continually assess and ensure that data is held in the most appropriate and secure way.

**What if I change my mind about taking part?**

You are free withdraw at any point of the project, without having to give a reason and this will not affect you in any way. You can withdraw data provided during interviews until 30^th^ July 2023, after which time this may not be possible due to processing and anonymisation of the data. Due to the nature of focus groups, it will not be possible to withdraw individual contributions after participation. If you choose to withdraw from the project we will not retain the information you have given thus far.

**How is the project funded?**

My PhD is funded by the Natural Environment Research Council (NERC) in the UK through the London NERC Doctoral Training Programme. You can learn more about my research on my [institutional page](https://www.kew.org/science/our-science/people/laura-kor).

**What if I have further questions?**

You can direct questions about the investigation, privacy, and handling of your personal data to Laura Kor [laura.kor@kcl.ac.uk](mailto:laura.kor@kcl.ac.uk).

You can contact the Ethics Committee of King’s College London: [rec@kcl.ac.uk](mailto:rec@kcl.ac.uk) if you have concerns about your rights as a participant.

**Please complete the consent form once you have read this information sheet and before the interview and return to:** [**laura.kor@kcl.ac.uk**](mailto:laura.kor@kcl.ac.uk) **or** [**l.kor@kew.org**](mailto:l.kor@kew.org)

**Appendix S3**: Interview guide for key informants on Important Plant Areas

***Interview details***

Date: ___________________________________

Survey number: ___________________________

***Participant information***

1. Consent form completed and returned? YES / NO

2. Name and email address (optional): ___________________________

3. What country are you based in? ________________________________

4. What sector do you work in?

1. Academic / Research
2. Conservation NGO
3. Government
4. Public body / Implementation agency
5. Industry
6. Other ________________________

5. What is your occupation?

***Topic 1: Experience and involvement with IPAs***

7. In what capacity have you been involved with IPAs? *Please select all relevant answers*

1. IPA identification in specific region or country
2. Development of IPA programme, guidelines, or approach globally or in specific region
3. Implementation of conservation and management related to IPAs
4. Interested in engaging with IPAs, but not yet done so
5. Other: _____________________________________________________________

8. Please could you add further details? (*e.g., if you were involved in identification, what elements were you involved with? Such as field data collection, biogeographical analyses, etc.)*

______________________________________________________________________

9. In what country or countries have you been involved with (or would like to be involved with) IPAs?

_____________________________________________________________________

***Topic 2: Approaches to and outcomes of IPA identification*** *(skip section if not relevant to participant, or only ask questions which are relevant to their experience and involvement)*

2.1. What was your primary aim in IPA identification? *(select all relevant answers, indicate rank if applicable)*

1. Research or academic interest
2. Inform conservation planning
3. Lead to conservation action
4. Engage stakeholders or increase awareness
5. No specific aim
6. Other_________________________________________________

11. Further details (*e.g., if to engage stakeholders, which stakeholders and why?*):

_________________________________________________________

12. Which criteria did you apply in your identification?

1. A: Threatened species
2. B: Botanical richness
3. C: Threatened habitats

13. Did you specifically consider socially, economically, and culturally important plant species (or useful plant species)?

1. Yes
2. No
3. Unsure

14. If yes, how? If no, why not and was this something that was or would be considered?

_________________________________________________________

15. What stakeholders were involved in your IPA identification process and how did you engage with them? (*e.g. researchers, NGOs, local communities, local or national authorities, etc.*)

_________________________________________________________

16. What methodologies and software did you use to assess data for IPA identification? (*e.g., no software, R, GIS, heuristic algorithms such as Marxan or Zonation, other)*

_________________________________________________________

17. What products did you produce, or do you plan to produce, related to IPA identification work? (*e.g., peer-reviewed articles, databases, reports, thesis, website, resources for local communities, species red listing, policy briefs, conference presentations, outreach events, media articles, etc.*)

_________________________________________________________

18. Is your IPA-related data publicly accessible? If so, where is it stored and available?

_________________________________________________________

***Topic 3: Perception of IPA programme***

*In this section, I will be asking questions which require an answer on a scale from 1 to 5, where 1 is “not effective” and 5 is “extremely effective”. Please explain the reasons for your answer if you wish to do so.*

20. Based on your experience, on a scale of 1 to 5:

A. How effective are IPAs in engaging in stakeholders working in botanical research and conservation?

1 [not effective] 2 3 4 5 [extremely effective]

Reason for your response (*if given*): ________________________________

B. How effective are IPAs in engaging other stakeholders (*e.g. local communities, decision makers, etc*.)?

1 [not effective] 2 3 4 5 [extremely effective]

Reason for your response (*if given*): ________________________________

C. How effective are the IPA criteria in identifying the most important sites for plant conservation at global or national scales?

1 [not effective] 2 3 4 5 [extremely effective]

Reason for your response (*if given*): ________________________________

D. How effective are the IPA criteria in accounting for local context and allowing incorporation of local ecological knowledge?

1 [not effective] 2 3 4 5 [extremely effective]

Reason for your response (*if given*): ________________________________

E. How effective are IPAs in enabling and achieving plant conservation action?

1 [not effective] 2 3 4 5 [extremely effective]

Reason for your response (*if given*): ________________________________

F. How much conservation value do IPAs add to other international area-based conservation initiatives (e.g. Key Biodiversity Areas (KBA) and Alliance for Zero Extinction (AZE) sites)?

1 [no additional value] 2 3 4 5 [vital as an independent initiative]

Reason for your response (*if given*): ________________________________

21. Please add any further thoughts on the topics above

_________________________________________________________

***Topic 4: IPA conservation action and community engagement***

22. Have the IPAs you have been involved with been incorporated into any conservation designations or processes in country? (*e.g. local, regional or national networks of protected areas*)

1. Yes
2. No
3. Unsure

23. Have you been involved with or know of conservation actions or community engagement undertaken due to IPA identification?

1. Yes
2. No

24. If yes, please describe the conservation intervention or community engagement undertaken.

*Potential prompts and follow-up questions: Who were the key stakeholders involved? How were sites for conservation chosen? What factors were important in enabling these interventions? What were the greatest challenges? What have the key outcomes been? Are interventions ongoing? Who leads and/or funds such conservation actions?*

_________________________________________________________

25. Are there any major opportunities or challenges you see for the IPA approach to support plant conservation?

_________________________________________________________

26. Do you have any further comments regarding IPAs or this work?

_________________________________________________________

27. Thank you so much for your time. Would you be happy to potentially be contacted by Plantlife to add to their list of IPA case studies?

YES / NO

**Appendix S4:** summary of results from negative binomial regression to model data collected from the systematic map of Important Plant Area records

glm.nb(Count ~ Continent * Year, data = year_cont)

Dispersion parameter (theta): 7.504

Residual deviance: 146.37 on 154 df

Significant relationships found:

- West Europe estimated 190% greater number of overall records than the reference group, Africa (*p* = 0.016)
- Number of records per year overall estimated to increase by 7.6% (*p* = 0.022)
- Number of records per year in West Europe estimated to decrease by 9.9% each year (*p* = 0.016)

**Appendix S5:** Number of records reviewed per year and continent (small random variation added to each point to enable all continents to be seen where data overlaps). Trend lines show the estimated count based on a negative binomial regression.


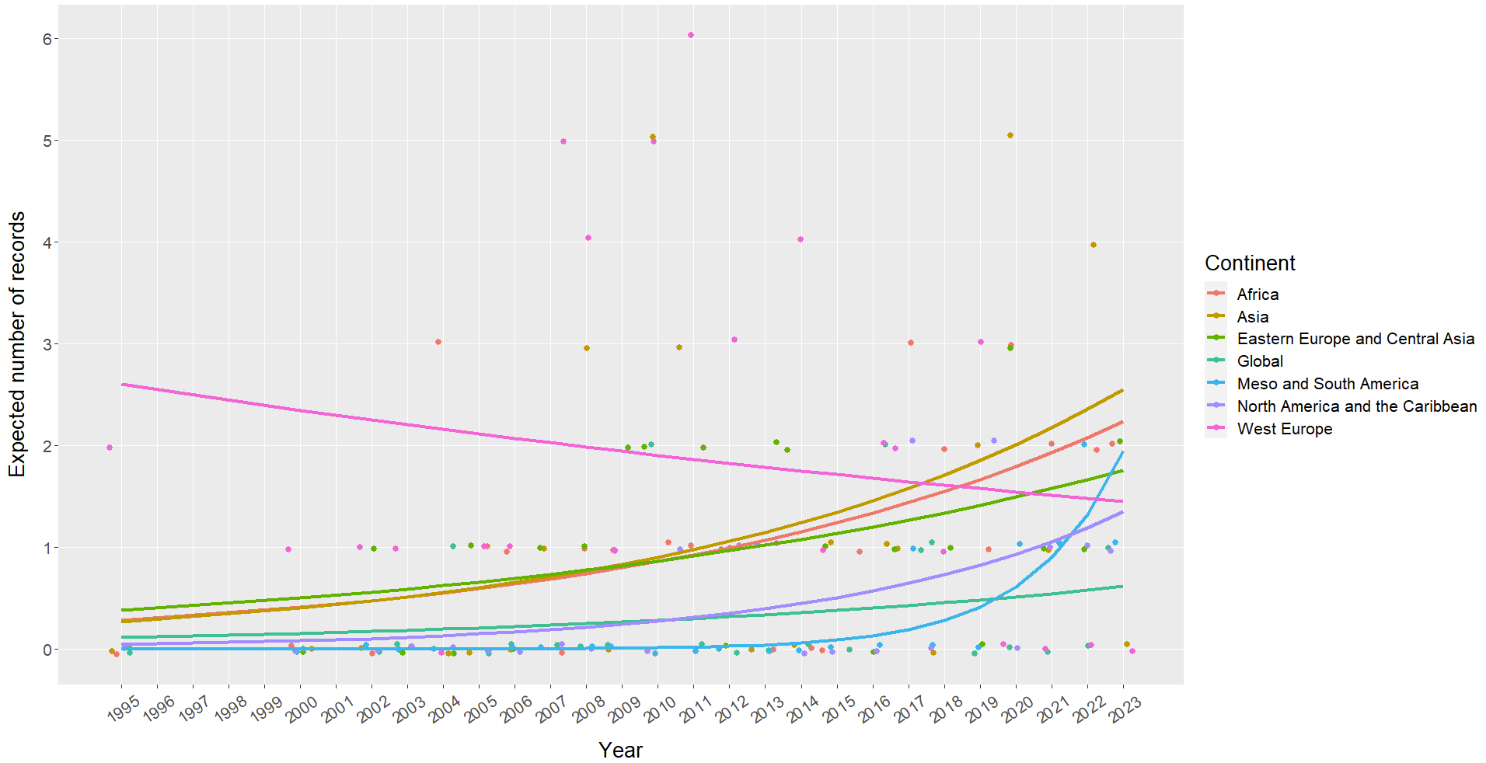

Supplement: Supplementary file 1 — Supporting Information [file COBI-39-e70013-s001.docx]
